# Unmet supportive care needs of young women with breast cancer in Chile during follow-up stage after treatment: A qualitative study

**DOI:** 10.1371/journal.pone.0330166

**Published:** 2025-08-13

**Authors:** Francisca Vezzani, Báltica Cabieses, Alexandra Obach, Sonia Torrealba, Iderta Carvajal

**Affiliations:** 1 Centro de Salud Global Intercultural, Facultad de Medicina Clínica Alemana, Facultad de Psicología, Universidad del Desarrollo, Santiago, Chile; 2 Department of Health Sciences, University of York, New York, United Kingdom; 3 Value and Access, Novartis Chile S.A., Santiago, Chile; Local Health Authority Caserta: Azienda Sanitaria Locale Caserta, ITALY

## Abstract

**Background:**

Breast cancer is a significant public health issue, with a rising incidence in young women who have more aggressive tumors and a poorer prognosis. In 2022, breast cancer accounted for 20.8% of all cancers in women in Chile, highlighting the urgent need for targeted research and support for young survivors. This study aims to explore the unmet supportive care needs of these young women during their follow-up period post-treatment, addressing gaps in existing literature and healthcare responses.

**Methods:**

Qualitative case study using semi-structured online interviews with 20 women who had a cancer diagnosis under 45 years of age. A thematic content analysis was conducted. Study approved by the Scientific Ethics Committee of the Universidad del Desarrollo, Chile.

**Results:**

Three primary areas of concern and unmet supportive care needs were identified during follow-up period: access to information, rehabilitation and integration, and mental health. These areas are deeply interconnected, and the absence of validation of these young women’s needs leads to a lack of adequate and comprehensive support from health professionals, deepening women’s sense of abandonment by the health system.

**Discussion and conclusion:**

The findings align with international literature, revealing that young women face unique challenges related to their life course. The lack of adequate support from healthcare professionals highlights the need for a multidisciplinary, person- and family-centred approach to care. It should address interlinked needs and advocate the involvement of patient organisations. This would enhance support and education regarding the needs of young women and their overall well-being during recovery process.

## Background

Breast cancer is a major public health challenge worldwide, as it is the leading cause of cancer-related death in women [1]. In Latin America, the incidence of breast cancer is increasing rapidly [2] and in Chile, breast cancer is the cancer with the highest incidence and mortality among women, with approximately 5,640 new cases and 1,775 deaths in 2022 [3]. With this increase in breast cancer incidence, the literature has shown that there has also been a global increase of 16% since the 1990s in young women [4], a group defined in recent literature as women under the age of 40 [4–8].

The characteristics of breast cancer in this age group are singular, with different risk factors, tumor biology and clinical outcomes [5]. They tend to be more aggressive tumors with more frequent recurrences, which would influence a worse prognosis [4,9,10]. In Chile, there is no specific data on the incidence of breast cancer in young women; however, it has been shown that the disease is more aggressive in this group, which translates into lower disease-free survival [10].

In turn, cancer treatment, in addition to physical and psychosocial needs, would cause late medical effects unique to this age group. This has been argued from the life course approach in which young women find themselves, given that they would have longer life spans ahead of them [5,11,12]. Among these effects, the occurrence of second primary cancers, fertility disorders, and cardiovascular and neurological complications have been documented. Younger women have also been identified as experiencing higher levels of distress and depression compared to older women who have had breast cancer [11–13].

These needs have been referred to in the literature as unmet needs or unmet supportive care needs. The definitions of these terms are still under discussion, but in general, unmet need has been seen as a critical indicator of access problems [14,15]. The concept of unmet supportive care needs that guides this study is used as a general term for needs that people perceive have not been adequately met and require additional support. These may be physical, emotional, informational, social, practical, spiritual, and others [16–18]. The importance of exploring and identifying these needs in breast cancer studies has been emphasised in order to achieve a person- and family-centred approach that effectively enables the provision of comprehensive healthcare management [16,19].

Despite the evidence collected on the effects experienced by young women in the follow-up and long survival period, the literature points out that this age group is underrepresented in research, which means that emerging supportive care needs are not necessarily adequately known or addressed [4,5,12,13,20]. Consequently, given the lack of studies on this issue, particularly in Chile, this study aims to explore young women’s perceptions of unmet supportive care needs during the follow-up period. This is the period following the end of the women’s treatment, during which they undergo regular check-ups.

To the best of our knowledge, this is the first study in Chile to explore this issue. It is hoped that it will provide updated evidence on a topic that has not yet been extensively researched in this country. The results provide valuable insights into the supportive care needs of young women who have completed their breast cancer treatment. They also help to identify priority issues that, despite emerging from a specific social and health context, exhibit common patterns with the international evidence on the unmet and supportive care needs of young women with breast cancer during the follow-up period.

## Materials and methods

This methods section follows the 32-item Consolidated Criteria for Reporting Qualitative Studies (COREQ) checklist, available in the supplementary material (S1 File).

### Study design

An exploratory and descriptive qualitative study was conducted using a multiple case study design, in which the researcher explored multiple bounded systems (i.e., several cases) through in-depth data collection [21,22]. Cases were defined and delimited according to the women’s health insurance, which may correspond to the private (Isapre), public (Fonasa) or armed forces health system.

### Recruitment and selection of participants

The sample of participants was based on theoretical and feasibility criteria. Within the theoretical definition, although recent literature defines the group of young women as those under 40 years of age, in this study we considered young women as those under 45 years of age, using as the main criterion that they were in the premenopausal period at the time of cancer diagnosis, as has been studied in other research [23]. Therefore, women who met the following criteria were selected: (i) aged 18 or older and 45 years or younger at diagnosis; (ii) breast cancer patient in Chile up to 10 years ago; (iii) treatment in the public or private system in Chile; (iv) access to the Internet for interviews in virtual format. The study did not include women who were in active treatment. Only women were included in the study due to its focus on the specific challenges faced by young women with breast cancer. This decision was based on literature showing that their medical, psychosocial and reproductive needs require further exploration and visibility.

A poster was created to recruit participants, with abbreviated information about the study and a link to an information sheet and an enrolment form. A foundation that supports women with breast cancer was then asked to disseminate the study to its contacts via email. In this way, women interested in participating signed up on the form and were contacted by one of the team’s researchers to arrange an interview date. As the interviews were carried out, new contacts were made. A total of 20 women participated in the study, and no further recruitment was done since data saturation was achieved. This means that the sufficiency of the number obtained is defined when the quality and quantity of the information collected responds to the study objective, observed when the information is repetitive and redundant and, therefore, does not contribute new elements [24]. This sample size is also consistent with existing literature, which indicates that data saturation in qualitative studies with relatively homogeneous populations and focused research aims is typically reached with 12–15 interviews [25–27].

### Data collection

Semi-structured individual interviews were conducted between January and May 2023, using a thematic interview script prepared by the researchers AO and BC, which addressed the dimensions of interest of the study, such as the perception of the diagnosis experience, perceived supportive care needs and barriers. The interviews were conducted by the researcher FV, who was previously trained and experienced in interviewing people with cancer. The interviews were conducted and recorded using the Zoom virtual platform and lasted approximately 60 minutes each. To guarantee the confidentiality of the interviews, participants signed an informed consent form beforehand, and the primary researcher used an exclusive Zoom account. The interviews were stopped once the team considered, through a preliminary analysis and codification process, that data saturation had been reached concerning the study’s objective.

### Data analysis

The interviews were transcribed verbatim into a Word document by a professional transcriptionist bound by confidentiality and reviewed by the team with the original audio to ensure the accuracy of each transcript. The transcriptionist anonymised sensitive information such as names, addresses and the names of health centres. Each transcript was also pseudonymised with a code before analysis. A thematic content analysis of the interview transcripts was carried out separately by FV, AO and BC using Atlas.Ti software, identifying patterns and themes based on the interview guidelines and derived from the data. The researchers first read the transcripts and conducted an open coding process by identifying concepts connected by similarities or differences within the raw data [24]. Subsequently, the concepts were grouped into codes and subcodes, which were then reviewed by the researchers, engaging in a consensus process regarding the established codes to agree on a final version (S1 Fig). The verbatim quotes included in this article were translated from Spanish by a native English speaker and reviewed by the authors to ensure that the translations maintained the original meaning.

### Qualitative scientific rigor

The study considered triangulation and theoretical-methodological adequacy as criteria of rigorousness [24,28]. Regarding triangulation, the information analysis was contrasted between the researchers of the team, who have backgrounds in different social sciences and health disciplines. This helps to reduce biases in the interpretation of the information. Regarding the theoretical-methodological adequacy, in the design phase of the study, the team ensured that the research problem had a logical relationship with the study’s methodology and theory to ensure the research’s coherence.

### Ethical considerations

The study was formulated under consideration of Chilean legislation to protect participants in health research. It was guided by the universal principles of scientific research (respect, beneficence, non-maleficence and justice). The research team ensured that participants received all the information about the study by providing them with an information sheet, and one of the researchers was available to answer any questions that arose to ensure their understanding and consequent voluntary participation in the study. Informed consent was signed digitally through the encrypted Alchemer platform. To safeguard the confidentiality of the participants, a code was assigned to each one. The study was approved by the accredited Scientific Ethical Committee of the Universidad del Desarrollo (#2022−96).

## Results

Participants in the study were diagnosed with breast cancer before or at the age of 45. Since the inclusion criteria were that they had been diagnosed with breast cancer in Chile up to 10 years ago, some of the women had been diagnosed at minimum 1 year ago (4 women) and others approximately 10 years ago (2 women). Four respondents were between 20 and 30 years of age at diagnosis (24,27,28,29), nine were between 31 and 41 years of age (31,33,34,36, two 37, two 39 and 41), and seven were between 42 and 51 years of age (43, two 44, four 45). Most interviewees resided in the Metropolitan Region, where the capital of Chile, Santiago, is located. Of those interviewed, 12 were treated in the public health system, 7 in the private health system and 1 in the armed forces health system. All of the interviewees were either beginning or undergoing the follow-up period. The sociodemographic characterization of the participants can be found in Table 1.

**Table 1:** Sociodemographic characterization of study participants.

Age of diagnosis	N	%
20-30 years	4	20%
31-41 years	9	45%
42-51 years	7	35%
**Total**	**20**	**100%**
**Region**	**N**	**%**
Metropolitan Region	18	90%
Other	2	10%
**Total**	**20**	**100%**
**Health insurance**	**N**	**%**
Public (Fonasa)	12	60%
Private (Isapre)	7	35%
Armed Forces	1	5%
**Total**	**20**	**100%**
**Educational level**	**N**	**%**
Primary/basic	0	0%
Secondary education	6	30%
Advanced technician	3	15%
Incomplete university	1	5%
University/Professional	10	50%
**Total**	**20**	**100%**

*Other regions refer to areas located north and south of Chile. The Metropolitan Region is considered the central region, where the capital is located.

The unmet supportive care needs reported by participants during the follow-up phase are grouped into three dimensions: a) need for information, b) need for rehabilitation and support in reintegration into work and daily activities, and c) need for mental health support.

### Information needs

In general, the interviewees describe the period following the end of treatment as one in which they feel abandoned by the health system, as it is a significant change for them to no longer have the support of the medical teams. One aspect that deepens the feeling of abandonment is access to information about the processes they go through in relation to the after-effects of treatment.

Respondents who had a mastectomy as part of their treatment report that they were not given information about the reconstruction process. While some share that it is not a problem for them, others find their self-esteem deeply affected. Thus, they arrive at the follow-up phase without clarity about the possibility of reconstruction and the physical requirements that must be met to undergo the medical procedure, such as, for example, the weight limit. In this sense, the participants highlight the need for information and alternatives regarding nutrition to reach the weight required by the doctors for the reconstruction procedure.

*“I intend to rebuild myself because I feel mutilated. I look deformed, I feel ugly and I have no one to tell me ‘look you have to do this’. I told the doctor that I wanted to reconstruct myself and he said ‘no, you have to lose 30 kg of weight and if you don’t lose it I can’t reconstruct you and, secondly, go to a bariatric surgeon because your only option is to have a bypass’. So I was expecting them to tell me, ‘here are the data, go to a nutritionist, see if with exercise, with some medication or with a diet plan you can lose a little weight’, but no, they send you right away to the last instance, because they want to get out of the issue quickly”. (E17, Public health system)*

It also highlights the need for information on treatments and their side effects in the medium and long term from a holistic point of view. In other words, health professionals should explain to patients how treatments and medications will affect them, considering not only physical aspects but also women’s mental health and how they can affect their relationship with their bodies.

The interviewees report that, just as during treatment, they need to monitor the side effects of the drugs they are given during the follow-up period and seek professional advice on how to deal with the after-effects. They point out the need for advice on the professionals to whom they should be referred to deal with the different after-effects of the treatments, such as a nutritionist or an ophthalmologist.

*“Chemotherapy affects you in everything, you have a cognitive loss, you have sight problems, you have dry mucous membranes, I mean all mucous membranes, I am talking about the nose, the mouth, the eyes, the gums, the vagina, the anus, you have constipation problems, physical problems, joint problems, all associated with chemotherapy and there is no integrated management, in other words the oncologist does not send you to a nutritionist, nor to an ophthalmologist, nor to a gynaecologist, nor to physiotherapy.” (E8, Private health system)*

Another relevant area where women interviewed say they need information is sexual and reproductive health. In general, they report that they are not given information about the effects of treatments on fertility, nor about the possibility of early menopause and the changes it entails both physically and psychologically. Few interviewees reported receiving information about fertility preservation alternatives. In contrast, most of the participants told that they were informed in an unempathetic way that they might become infertile, which initiates a fertility mourning process that is not accompanied by a psychosocial team.

*“They never talked to me about it, in fact, I was worried if I was already in the menopausal period and I went to a private gynaecologist, because I also had to know if I had to continue taking care of myself in terms of whether I was at risk of pregnancy or not”. (E20,Public health system)*

At the same time, the participants observed that sexuality during cancer is not addressed by health professionals, who do not inform them about how they can maintain a sexual life considering the side effects of the treatments, such as lack of sexual desire, dryness and pain. In addition to this, there is a lack of psychosocial support about the processes that women go through regarding their image and self-esteem, given that they have to recognize themselves in a new body, changed by the treatments.

*“The doctors don’t even mention the sexual part. It would be good to include sexual and couple issues, that there could be a therapist...because of the changes, I mean, I had a partial mastectomy, and I still feel that my breast is not the same, with drugs you have more pain, it swells more, so there is an issue that is not being addressed and it changes everything, it is much more complex to show yourself like that again and nobody addresses it.” (E11,Public health system)*

### Need for rehabilitation and support in the reintegration of work and daily activities

During the follow-up period, women begin a reintegration process, or not, to their daily activities and jobs. Given this, the women say that they experience the after-effects of the treatments without the support or guidance of a medical team as to which professionals they should be referred to treat the side effects. They report having cognitive problems, memory problems, attention problems, fatigue, and muscle weakness, among other aspects. These after-effects generate significant insecurities in women, causing them to question whether they will be able to carry out their daily activities as they did before the cancer diagnosis, and they fear that they will not be able to return to their professional workplaces.

*“‘Today I feel invalidated, because I have a loss of memory, of attention, and for the work I used to do, I don’t know if I could do it, I’m no good, because I wouldn’t do it well and for the same reason I haven’t gone back to work, because how am I going to go back to work if I don’t even feel capable of going out alone to the street or to my doctor or to my things.” (E19,Private health system)*

In this sense, they underline the need for a comprehensive rehabilitation program that considers multidisciplinary teams, where they are guided on how to deal with the side effects that appear after treatment. At the same time, they emphasize the importance of accompanying them in their reintegration into their daily and work activities. It is noteworthy that women approach patient organizations for rehabilitation, which offer various activities and instances of support. Among these, the women’s testimonies highlight the incorporation of sporting activities such as dancing or rowing, the latter to prevent the onset of lymphoedema after a mastectomy.

*“Rehabilitation is very scarce in breast cancer and it is something that has to be important. My arm was getting stiff, so that’s why I started exercising every day, moving my arm even if it hurts, just to be able to get higher up. They haven’t called me from the hospital to see a kinesiologist or anything, nothing, in fact.” (E2,Public health system)*

One aspect to note is that there is disagreement between women and health professionals as to when they should return to work. Participants report that professionals urge them to return to their daily routines as soon as possible, while they do not feel physically or mentally prepared to return to their activities. Consequently, they ask the doctors for an extension of their medical leave, which is often denied. Thus, the interviewees perceive that the after-effects they have to deal with, as well as their mental health, are not taken into consideration.

*“You go back to your old life and you feel horrible. I didn’t want to go back to work, but what people made me feel was that I didn’t want to go back to work because I was lazy and I wanted to enjoy the medical leave more, and it wasn’t about that, it was that my body couldn’t go back, you’re not ready to go back.” (E12,Private health system)*

### Mental health support needs

Based on the above, at this stage, the interviewees emphasize the need for a comprehensive approach to the therapeutic process, incorporating mental health support from the diagnosis of the disease to the entire process following the end of treatment. Among the aspects that affect women’s mental health is the process of recognizing their bodies and state of health after treatment, where, although they have recovered from cancer, they are faced with new ailments and sequelae. In this sense, the modification of body image and the effects on self-esteem also affect women’s sex lives.

*“But more than that, it’s feeling like I’m getting old, not on the outside, on the inside, you feel like...menopause reminds me that I’m not healthy. I feel like a little old lady, I have no sexual desire, now I need to go around with my lubricant because if I don’t, I can’t.” (E12,Private health system)*

Furthermore, women often question their abilities and skills in light of the effects of treatment. Participants also report living in constant fear of recurrence or the appearance of a secondary cancer, which keeps them alert to any strange signs or symptoms in their body.

*“‘I feel that there is neglect; psychological attention should be every three months because one feels many things, one is very persecuted by cancer. First of all, there has to be weekly psychological therapy, for at least a year I think. And if it is not a psychologist, a multidisciplinary team for the person who remains post-cancer because many things remain in the head, there is a problem that also occurs at the cerebral level, I am like very distracted, I have difficulties to remember, I have forgotten many things’.” (E5,Public health system)*

In light of the above, women share that their mental health is profoundly affected and recognize that they have begun to suffer from depression and anxiety during this time. As a result, they emphasize the need for the health system to provide ongoing psychological support throughout the process and to continue to do so at regular intervals after treatment has ended. In the absence of such psychosocial support, as experienced by the interviewees, several participants resorted to the support of civil society organizations. These are crucial in accompanying, guiding and educating patients on several unmet needs. For example, some provide free psychological attention or offer support groups, inform about the side effects of treatments, and conduct workshops on self-esteem, image and sexuality, among others.

*“I asked the doctor, is there psychological support for this process? He said no. I feel that it is very important because if it weren’t for the different organizations that you get to know...you go to hell.” (E17,Public health system)*

## Discussion

This study explores perceived unmet supportive care needs experienced by young women during the follow-up period after breast cancer treatment in Chile. The primary needs identified relate to a) access to information, b) rehabilitation and reintegration into activities, and c) mental health.

These findings are consistent with those reported in the international literature, highlighting the limited information available on the impact of treatments on physical and psychological well-being [4,29–31]. The results of the latter align with international studies on mental health consequences, which have shown that young women experience feelings of uncertainty and liminality, which are generated by the fear of recurrence. This is also linked to their relationship with their body, where their self-esteem is affected, which in turn affects their sexual and relationship dissatisfaction [13,32–36].

In this context, the lack of clear information regarding breast reconstruction options and the physical requirements for such procedures –such as weight limits—further contributes to distress among some women who underwent mastectomy. While not a concern shared by all, this gap in information affected the emotional wellbeing and body image of several participants. These findings underscore the need for anticipatory and holistic guidance, including early conversations about reconstruction and nutritional counselling, in order to reduce uncertainty and support psychological adjustment during follow-up phase.

In addition to the above, the results show that this age group faces significant insecurities regarding the reintegration into their daily activities, especially into the labor market, due to the side effects of the treatments, such as memory loss. This is largely supported by the literature [13,37,38]. It is observed that these three unmet supportive care needs perceived by young women are strongly interrelated. Therefore, it has been emphasized that health professionals should incorporate a patient- and family-centered, psychosocial approach to the follow-up phase and not only focus on controlling possible recidivism [39]. In this regard, it has been argued that health professionals must recognize and validate women’s needs and concerns to develop interventions relevant to their life course [36,40].

Consequently, the importance of creating multidisciplinary teams to accompany women during the follow-up phase, including doctors, nurses, psychologists, social workers, occupational therapists, and nutritionists, among others, has been established. At the same time, the importance of including patient organisations in these teams is emphasised, given the vital role they play in providing information, support, and enhancing self-esteem [12,31,32,41,42]. Regarding women’s sexual health concerns, the literature supports the fact that health professionals are not prepared to address this issue with women, so it is necessary to generate a model that allows professionals to educate about sexual health from a holistic approach [36]. At the same time, it has been recommended that women’s partners should be involved in and educated about the diagnosis, treatment and follow-up processes, so that they are aware of the changes and effects that the treatments will have on their partners [36,43].

As for reintegration into daily activities and workspaces, one study recommends that medical and psychosocial teams support women requesting necessary work adaptations [13,37,38]. It is therefore suggested that the transition of women from active treatment to the follow-up stage represents a critical moment in which women’s needs must be addressed comprehensively [39]. However, this also requires a consensus on what it means to be a survivor, given the many definitions and discussions around the concept and the time period under which follow-up is defined [44].

The results contribute to the knowledge of unmet supportive care needs among young women during the follow-up period. Still, the limitations of this study should be taken into consideration for future research. More than half of the participants had a university or professional education, which may have influenced their ability to articulate their needs, navigate the health system and seek information or support. This may have shaped the prominence of specific themes, particularly those related to informational needs and advocacy. Consequently, the transferability of the findings to women with lower levels of formal education, who may encounter different obstacles, may be limited. Future studies should include a more diverse range of educational backgrounds to capture the variety of survivorship experiences. Other aspects not covered by this study could also be explored, such as researching the needs of people with different breast cancer subtypes in rural and urban areas, and of varying gender identities and socioeconomic statuses. Furthermore, the experience of men with breast cancer should be addressed.

Among the implications for practice, this study provides information for health services to anticipate and manage supportive care needs, where international evidence suggests that innovative out-of-hospital care strategies can be developed to avoid over-medicalization of women at the end of treatment [34]. Therefore, knowledge of unmet supportive care needs provides an opportunity for health services to address the post-treatment period through innovative strategies, using a comprehensive and person- and family-centred approach that respects the specificities of the individual and their significant others. Some innovative strategies that could be considered include tele-survivorship visits, which enable structured virtual follow-ups with multidisciplinary teams and increase accessibility for women balancing work and caregiving responsibilities [45]. Mobile health apps tailored for young cancer survivors can support symptom tracking, emotional well-being and peer interaction [46]. Additionally, peer-led navigation programmes, in which trained survivors support women through the post-treatment phase, have demonstrated potential in enhancing emotional support and facilitating navigation of the healthcare system [47]. Implementing these approaches in collaboration with community-based organisations may improve the continuity and personalisation of follow-up care.

## Conclusions

This qualitative study provides evidence of the unmet supportive care needs of young women in Chile during the follow-up period after breast cancer treatment. Participants reported significant gaps in three interrelated areas: access to timely and clear information; access to rehabilitation services to support a return to work and social roles; and sustained psychological support that considers their age. Without systematic addressing of these needs by the health system, women experience a sense of abandonment and must rely on patient organisations and informal networks for guidance. These findings emphasise the importance of transitioning to innovative, integrated, person- and family-centred care models that incorporate structured survivorship follow-up strategies linked to community services. They also emphasise the importance of incorporating survivors’ views in the design and implementation of long-term oncology care policies and practices.

## Supporting information

S1 File COREQ checklist. (PDF)

S1 Fig Coding tree. (TIF)

S2 File Interview guide. (PDF)

S3 File Research team characteristics and qualifications. (PDF)
