# Supplementary material for: Unmet supportive care needs of young women with breast cancer in Chile during follow-up stage after treatment: A qualitative study
Source: PLoS One. 2025 Aug 13;20(8):e0330166. doi: 10.1371/journal.pone.0330166 (PMC12349065; doi:10.1371/journal.pone.0330166)
Supplement: S1 File — (PDF) [file pone.0330166.s001.pdf]

Supplementary material: COREQ Checklist

Unmet needs of young women with breast cancer in Chile during follow-up: a qualitative study

Francisca Vezzani[1], Báltica Cabieses[1,2], Alexandra Obach[1], Sonia Torrealba[3], Iderta Carvajal[3]

1. Centro de Salud Global Intercultural, Facultad de Medicina Clínica Alemana, Facultad de Psicología, Universidad del Desarrollo, Santiago, Chile.
2. Department of Health Sciences, University of York, UK.
3. Value & Access, Novartis Chile S.A.

Supplementary File 1: CORE-Q checklist of the research *"Unmet needs of young women with breast cancer in Chile during follow-up: a qualitative study."*

Item	Topic	Guide Question	Descriptions	Report on (page/table)
**Domain 1: Research team and reflexivity**				
Personal Characteristics				
1	Interviewer/facilitator	Which author/s conducted the interview?	The author that conducted the interviews is FV. This is also presented in the manuscript.	Page 5.
2	Credentials	What were the researcher's credentials?	The credentials of the researchers who participated in this study are described in the manuscript in general and in detail in the Supplementary material.	Page 6 and<br><br>Supplementary material N°3.
3	Occupation	What was their occupation at the time of the study?	The occupations of the researchers who participated in this study are described in the manuscript in general and in detail in the Supplementary material.	Supplementary material N°3.

4	Gender	Was the researcher male or female?	The researcher who conducted the interviews is a female. The gender and initials of all the researchers are described in Supplementary Material.	Supplementary material N°3.
5	Experience and training	What experience or training did the researcher have?	The experience and training of the researcher who conducted the interviews are described in a general way in the manuscript, and the experience of the rest of the team is described in detail in Supplementary Material.	Page 5 and<br><br>Supplementary material N°3.
Relationship with participants				
6	Relationship established	Was a relationship established before study commencement?	The researchers informed all potential participants of the study objectives and their right to decline participation or withdraw consent at any research stage. Prior to the study, all participants had the opportunity to ask questions to the researcher or study coordinator by mail or phone. All participants provided online informed consent to participate in the study.	Page 4-5.
7	Participant knowledge of the interviewer	What did the participants know about the researcher? (e.g., personal goals, reasons for doing the research)	Potential participants knew the name of the principal researcher, place of work and reason for doing the research. Also, they were informed of the reason and importance of this research. This information was presented in an information sheet given	Page 4-5.

			to each participant before participation.	
8	Interviewer characteristics	What characteristics were reported about the interviewer/facilitator? E.g., bias, assumptions, reasons and interest in the research topic.	Potential participants were informed of the interviewer's name and place of work. The reasons and interest in the research topic were presented in the information sheet given to each participant before participation.	Page 4-5.
**Domain 2: Study design**				
Theoretical framework				
9	Methodological orientation and theory	What methodological orientation was stated to underpin the study? E.g., grounded theory, discourse analysis, ethnography, phenomenology, content analysis.	We employed thematic content analysis in this study.	Page 5
Participant selection				
10	Sampling	How were participants selected? E.g., purposive, convenience, consecutive, snowball.	The sample of participants was based on theoretical and feasibility criteria. Therefore, to the selected participants based on theoretical criteria, feasibility criteria were applied according to their availability and willingness to participate.	Page 4-5
11	Method of approach	How were participants approached? E.g., face-to-face, telephone, mail, email.	Participants were recruited by mail and then contacted by phone.	Page 5
12	Sample size	How many participants were in the study?	There was a total of 20 participants in the study.	Page 5 and Table 1.

13	Non-participation	How many people refused to participate or dropped out? Reasons?	Participation in the in-depth interviews was voluntary, and no participants declined to participate.	
Setting				
14	Setting of data collection	Where was the data collected? E.g., home, clinic, workplace.	The data was collected online.	Page 5.
15	Presence of non-participant	Was anyone else present besides the participants and researchers?	No one else was present besides the researcher and participants.	
16	Description of sample	What are the important characteristics of the sample? E.g., demographic data, date.	Participants' sociodemographic data is described on Page 7 in Table 1.	Page 7, Table 1.
Data collection				
17	Interview guide	Were questions, prompts, guides provided by the authors? Was it pilot tested?	The Interview guide is provided by the research team in supplementary file N°2.	Supplementary file N°2.
18	Repeat interviews	Were repeat interviews carried out? If yes, how many?	There were no repeat interviews carried out in this study.	
19	Audio/visual recording	Did the research use audio or visual recording to collect the data?	Interviews were audio recorded through the Zoom platform.	Page 5.
20	Field notes	Were field notes made during and/or after the interview or focus group?	n/a	
21	Duration	What was the duration of the interview or focus group?	The interviews had an average duration of 60 minutes.	Page 5.
22	Data saturation	Was data saturation discussed?	The information saturation was assessed after interim data analysis by the research team.	Page 5.

23	Transcripts returned	Were transcripts returned to participants for comment and/or correction?	Transcripts were not returned to participants, but they were checked for accuracy against the original audio by more than one research team member.	Page 5.
Data analysis				
24	Number of data coders	How many data coders coded the data?	3 researchers coded the data.	Page 5
25	Description of the coding tree	Did authors provide a description of the coding tree or codebook?	Yes, it is in a figure in page 6.	Page 6.
26	Derivation of themes	Were themes identified in advance or derived from the data?	Themes were identified in advance, and emergent themes were identified through analysis.	Page 5
27	Software	What software, if applicable, was used to manage data	Atlas.ti	Page 5
28	Participant checking	Did participants provide feedback on the findings?	Participants received the findings via e-mail. Participants did not give feedback.	
Reporting				
29	Quotations presented	Were participant quotations presented to illustrate the themes/findings? Was each quotation identified? e.g., participant number.	Representative quotes and the respective code illustrate the findings in the results section.	Pages: 8 to 13.
30	Data and findings consistent	Was there consistency between the data presented and the findings?	We demonstrate consistency between the data presented in the results section and the interpretation of findings in the discussion section.	Pages: 8 to 15.
31	Clarity of major themes	Were major themes clearly presented in the findings?	We presented all major themes according to the objective of the study.	

32	Clarity of minor themes	Is there a description of diverse cases or discussion of minor themes?	We provided a diversity of quotes based on the different participants.	Pages: 8 to 13.
