# Supplementary figures and images for: Unmet supportive care needs of young women with breast cancer in Chile during follow-up stage after treatment: A qualitative study
Source: PLoS One. 2025 Aug 13;20(8):e0330166. doi: 10.1371/journal.pone.0330166 (PMC12349065; doi:10.1371/journal.pone.0330166)

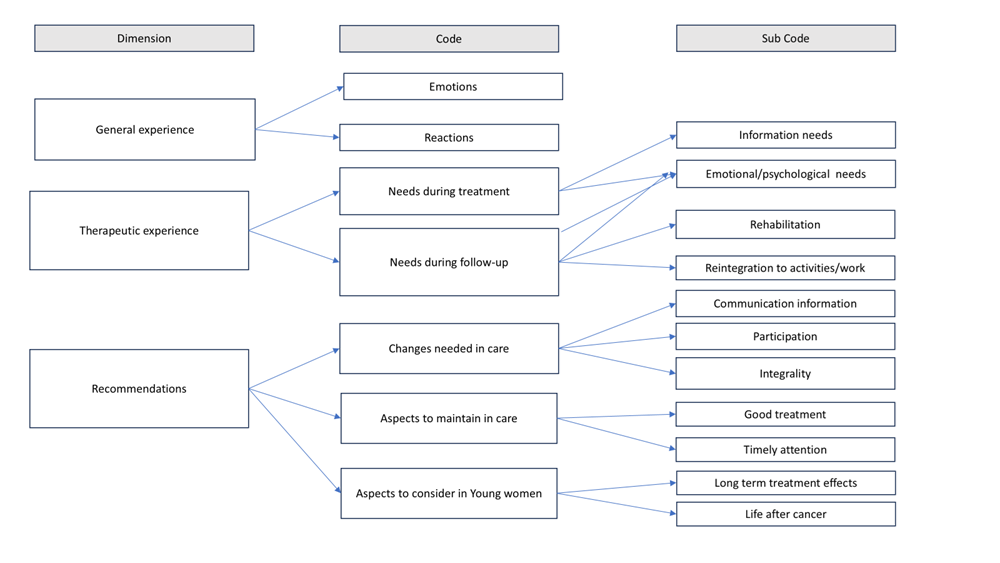

Supplement: S1 Fig — (TIF) [file pone.0330166.s002.tif]
