# Supplementary material for: Unmet supportive care needs of young women with breast cancer in Chile during follow-up stage after treatment: A qualitative study
Source: PLoS One. 2025 Aug 13;20(8):e0330166. doi: 10.1371/journal.pone.0330166 (PMC12349065; doi:10.1371/journal.pone.0330166)
Supplement: S2 File — (PDF) [file pone.0330166.s003.pdf]

Supplementary material: Interview guide

Unmet needs of young women with breast cancer in Chile during follow-up: a qualitative study

Francisca Vezzani[1], Báltica Cabieses[1,2], Alexandra Obach[1], Sonia Torrealba[3], Iderta Carvajal[3]

1. Centro de Salud Global Intercultural, Facultad de Medicina Clínica Alemana, Facultad de Psicología, Universidad del Desarrollo, Santiago, Chile.
2. Department of Health Sciences, University of York, UK.
3. Value & Access, Novartis Chile S.A.

Supplementary File 2: Interview Guide for interviews.

**Instructions to the interviewer**
1. Apply the interview script only once the online informed consent has been signed.<br><br>2. Have a backup audio recorder handy if the main one fails.<br><br>3. Observe the participant's state, stopping whenever necessary and checking if he/she wants to continue participating.
**Opening sentence**
Welcome participant and introduce yourself.<br><br>Explain the purpose and process of the in-depth interview.<br><br>Explain the presence and purpose of recording equipment.
**Demographic data from the interviewee**
Ask about name, age, sex, region of residence, education level, profession/activity
**Discussion guidelines**
General experience

Can you tell me a little bit about yourself and what your breast cancer diagnosis was like?<br><br>*What was your reaction to receiving the diagnosis, how did you deal with it at the time?*
Therapeutic Experience<br><br>*Throughout your therapeutic journey, what specific needs did you consider important (emotional support, medical information, etc.)?*<br><br>*How did they help you meet those needs?*<br><br>*After completing your treatment, how was your experience during the follow-up period?*<br><br>*What kind of support did you continue to receive?*<br><br>*Where do you feel support is lacking?*
Recommendations<br><br>*What would you change in your experience with breast cancer care?*<br><br>*What changes would you recommend to improve care for young women with breast cancer?*<br><br>*On the other hand, are there things that you think should be kept as they are in the care process?*<br><br>*What services or supports do you consider essential and worked well for you?*<br><br>***Final message***<br><br>*Is there anything else you would like to share about your experience or any message you would like to pass on to other young women in a similar situation?*
