# Supplementary material for: Unmet supportive care needs of young women with breast cancer in Chile during follow-up stage after treatment: A qualitative study
Source: PLoS One. 2025 Aug 13;20(8):e0330166. doi: 10.1371/journal.pone.0330166 (PMC12349065; doi:10.1371/journal.pone.0330166)
Supplement: S3 File — (PDF) [file pone.0330166.s004.pdf]

Supplementary material: Research team characteristics and qualifications

Unmet needs of young women with breast cancer in Chile during follow-up: a qualitative study

Francisca Vezzani[1], Báltica Cabieses[1,2], Alexandra Obach[1], Sonia Torrealba[3], Iderta Carvajal[3]

1. Centro de Salud Global Intercultural, Facultad de Medicina Clínica Alemana, Facultad de Psicología, Universidad del Desarrollo, Santiago, Chile.
2. Department of Health Sciences, University of York, UK.
3. Value & Access, Novartis Chile S.A.

Supplementary File 3: Research Team Characteristics and Qualifications

**Author**	**Characteristics and Qualifications**
BC	Female Nurse-midwife (2002, PUC Chile), diploma in university teaching (2004, PUC Chile), Master in Epidemiology (2008, PUC Chile) and PhD in Health Sciences (mention in social epidemiology) from the University of York, England (2011). Professor of social epidemiology and director of the Center of Global Intercultural Health at the Faculty of Medicine Universidad del Desarrollo. Visiting scholar at the Department of Health Sciences at the University of York. Member of Lancet Migration for Latin America, board member of the Chilean network of research on health and migration RECHISAM. Former Vice-President of the Chilean Society of Epidemiology between the years 2014-2017. National and international consultant of health equity, health of migrants and implementation science in socially and culturally diverse communities. Advisor to WHO, PAHO, the Ministry of Health and the Ministry of Social Development of Chile, and to various public and private institutions. Research lines: social inequities in health (2005 to date), health of international migrants (2008 to date) and participation of patients in decision-making on health coverage (2016 to date). Editor of 8 academic books and over 20 publicly available research reports and policy briefs. Participation in more than 60 research projects in Chile and abroad and has more than 170 scientific publications.
AO	Female Social Anthropologist. She has a master's degree in Gender and Cultural Studies from the University of Chile, and a doctorate in Social and Cultural Anthropology from the University of Barcelona. Associate Professor and Executive Director of the Center for Global Intercultural Health (CeSGI), Universidad del Desarrollo. She is an expert in health, gender and intercultural issues. Research focuses on health anthropology, adolescent and youth sexual and reproductive health, patient participation in health decision-making, and qualitative methodologies. Principal investigator of various research projects,

	including: Fonis Project #SA15I20040; Fonis Project #SA19I0091; Fondecyt #11190701.
FV	Female, Social Anthropologist, MSc (c) in Public Health (Universidad de Chile). Diploma in Quantitative Methodologies (Universidad Diego Portales), and diploma in Formulation and Evaluation of Social Projects focused on the territory and the community (Pontificia Universidad Católica de Chile). Researcher in Center for Global Intercultural Health (CeSGI), ICIM, Universidad del Desarrollo. Research interests include anthropology and health, public health, inequities, patient participation, cancer and rare diseases.
ST	Female, Commercial Engineer (Pontificia Universidad Católica de Chile). Master in Government and Public Management (Universidad de Chile). Diploma in International Financial Reporting Standards and Diploma in Senior Management in Networks and Health Establishments (Universidad de Chile). Health Care System & Policy Manager in Novartis Chile S.A.
IC	Female, Veterinarian (Universidad de Chile). Diploma in Evidence-Based Medicine and Public Health (Universidad de los Andes). Diploma in Management of Public and Private Health Organizations (Universidad de los Andes). Health Care System Evidence & Pricing Manager in Novartis Chile S.A.
